# New and Updated Features in Phenix for Macromolecular Structure Determination

**DOI:** 10.1063/4.0001028

**Published:** 2025-10-27

**Authors:** Billy K Poon

**Affiliations:** Lawrence Berkeley National Laboratory, Berkeley, CA, USA

## Abstract

In this presentation, we will highlight the newest developments in Phenix. Our newest release, Phenix 2.0, includes the following new features: more accurate restraints for ligands in situ using quantum mechanical calculations, quantum refinement for proteins using machine learning, improved tools for accessing RCSB web services (such as the PDB validation report), and simplified usage of reference models for reciprocal space refinement which is especially useful at low resolution. We will describe how to use these new tools, discuss other updates in Phenix, and explore future developments.

